# Points from the Quarterly Report

**Published:** 1916-06-10

**Authors:** 


### THE LONDON HOSPITAL.

### Points from the Quarterly Report.

THE report presented to the meeting of the Quarterly Court held on June 7 states that owing to the continuance of the war the running of the London Hospital has been more difficult than ever, although the board think it probable that the most difficult time is still to come, especially as regards the workers at the hospital, as under the Compulsion Act the majority of the remaining medical and lay staff of military age will be called up by the end of July.

The financial position created by the great rise in the cost of all articles of food, as well as drugs, dressings, and everything in daily use, is such as to cause the committee some anxious thought. A sub-committee consisting of three of the members of the house committee, together with a representative of the surgical and one of the medical side, is thoroughly examining the details of the working of the whole hospital, with a view to deciding whether it is in any way possible to effect any economy.

The staff difficulty will be felt particularly in the out-patient department, which is working at great pressure. The great majority of the clerks will have left by the end of July, and their places will be filled by ladies, who will take some time to get accustomed to somewhat technical work. Similarly, some of the dispensers are being called up, and their places filled by lady dispensers.

### A British Catgut Supply.

An interesting situation has arisen through the shortage of catgut, which is largely used in operations. The supply formerly came exclusively from Saxony, and a problem arose as to restocking, but fortunately an Edinburgh chemist has started a factory for its manufacture, where, the sanitary and antiseptic conditions having been found most satisfactory, a fresh supply was at once secured. It may be hoped for the future to obtain this indispensable article from our own country, instead of having to depend for its supply on Germany.

### A Tribute to Tradesmen.

The hospital expresses its acknowledgment to the various firms who have supplied necessaries during a difficult time, and to them official letters of thanks have been written; so valuable has been the service rendered in some cases that several life governorships have been conferred.

### The New Wards.

The St. Anthony Wards, so generously presented by the Grocers' Company, have now been opened, and already 156 patients suffering from syphilis have been treated with conspicuous success. Recently the Right Hon. Walter Long, the President of the Local Government Board, who was accompanied by Dr. Newsholme, Chief Medical Officer of the Board, visited the wards, and expressed himself as most interested and pleased with the work which is being done in this direction.

Lord Knutsford, who has been making satisfactory progress since his serious accident, has left Scotland, where he was sent for a period of convalescence, and is now in Wales, whence he is expected to return at the beginning of July, when the board hope that his most invaluable work for this institution will be renewed.

The matron, Miss E. C. E. Luckes, has also made a good recovery, and has been back at work for the last few weeks after an absence of over three months.

### Birthday Honours.

The London Hospital was represented in the King's Birthday Honours List by the senior surgeon of the dental staff, Mr. F. M. Farmer, who received the honour of knighthood in recognition of work during the present war and the South African campaign. Sister Currie (Miss Martha Beatley) and Sister Charlotte (Miss Frances Coombe) have received the distinction of being decorated with the Second Class Royal Red Cross. Seven other former London nurses, who are now working in various hospitals for the benefit of the wounded, received the same decoration.

Messrs. Bryant and May have made a handsome gift to the hospital in the shape of an ambulance.

